# Sustainable Use of Greek Herbs By-Products, as an Alternative Source of Biologically Active Ingredients for Innovative Products

**DOI:** 10.3389/fnut.2022.867666

**Published:** 2022-04-07

**Authors:** Evanthia Dina, Argyro Vontzalidou, Antigoni Cheilari, Panagiotis Bagatzounis, Eftyxia Agapidou, Ilias Giannenas, Katerina Grigoriadou, Nektarios Aligiannis

**Affiliations:** ^1^Department of Pharmacognosy and Natural Products Chemistry, Faculty of Pharmacy, National and Kapodistrian University of Athens, Panepistimiopolis Zografou, Athens, Greece; ^2^Bagatzounis & Sons S.A, Kozani, Greece; ^3^ELVIZ Hellenic Feedstuff Industry S.A., Plati-Imathia, Greece; ^4^Laboratory of Nutrition, School of Veterinary Medicine, Faculty of Health Sciences, Aristotle University of Thessaloniki, Thessaloniki, Greece; ^5^Institute of Plant Breeding and Genetic Resources, Hellenic Agricultural Organization – DEMETER, Thessaloniki, Greece

**Keywords:** MAPs' by-products, *Origanum vulgare* subsp. *hirtum*, *Sideritis scardica*, *Thymus vulgaris*, *Matricaria recutita*

## Abstract

The processing of medicinal and aromatic plants (MAPs) results in the production of a significant amount of plant by-products; herbal material of inferior quality and/or unusable plant parts that are not commercially exploitable. An extensive study of Greek native species was performed toward the production of innovative bioactive products using as raw materials the by-products obtained from the processing of cultivated MAPs. *Origanum vulgare* subsp. *hirtum* (oregano), *Sideritis scardica* (Greek mountain tea), *Thymus vulgaris* (thyme), and *Matricaria recutita* (chamomile) were selected due to their wide use for the preparation of beverages and culinary purposes. The determination of the percentage of the post-harvest processing by-products was performed for a 3 years period (2018–2020). Results showed that by-products derived from the above-mentioned species' processing constitute 64% (thyme), 54% (oregano), 37% (Greek mountain tea), and 24% (chamomile) of the total processed mass. To value the by-products as a potent source of bioactive ingredients, superior and inferior quality herbal material of the aforementioned plant species were extracted by an ultrasonic assisted extraction method. Hydroalcoholic extracts were chemically investigated using high-performance thin layer chromatography (HPTLC) and liquid chromatography-mass spectrometry (LC-MS) techniques. In addition, their free radical scavenging activity and total phenolic content (TPC) were estimated. Based on the results, herbs by-products revealed similar chemical content to the superior herbal material by the means of HPTLC and LC-MS analysis. In addition, strong free radical scavenging related to a high phenolic content was detected in the case of thyme, oregano, and Greek mountain tea. Moreover, the gas chromatography-mass spectrometry (GC-MS) analyses of the essential oils (EOs) of oregano and thyme by-products revealed the presence of carvacrol, thymol, γ-terpinene, and *p*-cymene among the major constituents. Finally, the LC-MS analyses of aqueous extracts of Greek mountain tea and chamomile by-products led to the identification of several bioactive compounds, such as flavonoids and phenylpropanoids. Overall, the presence of bioactive constituents in by-products, such as terpenes, phenolic compounds, and flavonoids underly their potent use as food antimicrobial and antioxidant additives, in the preparation of high added-value products, such as enriched aromatic edible oils, and innovative herbal teas, such as instant beverages.

## Introduction

The processing procedure of medicinal and aromatic plants (MAPs) results in a significant amount of by-products, such as hydrolates and solid residues from the essential oils (EOs) process (1), or post-harvest by-products, such as branches and leaves of inferior quality (2), that are non-commercially acceptable (3). These residual biomasses are the potential sources of bioactive compounds, since they contain the same ingredients and properties as the final product (4, 5). Until now, these materials were treated as waste and disposed improperly to the fields or used as a burning material. However, MAPs by-products are a reservoir of valuable metabolites with important biological properties, which could add special value to the final products (6).

Oregano (*Origanum* spp.) and thyme (*Thymus* spp.) are among the world's most valued aromatic plants, not only for culinary purposes, but also for their EOs. The EO of thyme has a milder odor compared with oregano, mainly because it contains thymol in larger quantities compared with its isomer carvacrol, with major antimicrobial, antioxidant, and anti-inflammatory properties (7–9). Greek mountain tea (*Sideritis* spp.) possess antioxidant, anti-inflammatory, and antimicrobial properties (10, 11). Chamomile (*Matricaria recutita* L.) is a medicinal plant used traditionally as a mild sedative and to treat gastrointestinal problems. It has been shown to possess anti-inflammatory and antiviral properties (12, 13). These herbs possess the aforementioned biological properties due to the occurrence of several secondary metabolites, such as phenolic constituents, terpenoids, and flavonoids, in their extracts. Due to their rich chemical content, these herbs are the most frequent cultivated species in Greece, to produce EOs or they are sold as raw botanicals for the preparation of herbal teas. However, during the post-harvest processing for the selection of the marketed material, significant amount of herbal residues is produced. According to the farmers, limited part of these residues is utilized as fertilizer, but the massive production is rejected in the fields polluting the environment. Up to now, several studies have proven the presence of bioactive constituents in the selected plants by-products (14, 15). Toward this direction, the remaining herbal and/or hydrodistillation by-products constitute a promising source of bioactive compounds with potential applications in pharmaceutical, cosmeceutical, and food supplements industries. Hence, in continuation of our research efforts on MAPs by-products (16), the aim of this study was to assess in a systematic way, the amount of by-products generated after the harvesting and processing procedure of four Greek MAPs (thyme, oregano, Greek mountain tea, and chamomile). Moreover, the aim was to characterize the chemical profile and value the by-products as a potent source of bioactive ingredients for the production of innovative foods additives and high-added value products, such as instant beverages or enriched aromatic olive oils.

## Materials and Methods

Analytical grade methanol (MeOH) for extraction, as well as acetonitrile (ACN), acetic acid (A.A), sulfuric acid (H_2_SO_4_), and vanillin for high-performance thin layer chromatography (HPTLC) analysis and ethanol for bioassays were purchased from Merck (Merck, Darmstadt, Germany). For free radical scavenging and total phenolic content assays, Folin–Ciocalteu solution, dimethylsulfoxide (DMSO), sodium carbonate (Na_2_CO_3_), gallic acid, and 2,2-diphenyl-1-picrylhydrazyl (DPPH) were purchased from Sigma-Aldrich (Sigma-Aldrich, Steinheim, Germany).

### Plant Material

The by–products production by the processing of four Greek cultivated species, *Origanum vulgare* subsp. *hirtum* L. (oregano, IPEN (International Plant Exchange Network) accession number GR-1-BBGK- 03,2107), *Sideritis scardica* L. (Greek mountain tea, IPEN accession number GR-1-BBGK- 13,5769), *Thymus vulgaris* L. var Varico 3 (thyme), and *Matricaria recutita* L. var Banatsa (chamomile, IPEN accession number GR-1-BBGK- 21,1) was studied. Plant propagating material was originated by mother plants maintained *ex-situ*, at the collection of Balkan Botanic Garden of Kroussia (41°05′44.3″N 23°06′33.7″E) of the Institute of Plant Breeding and Genetic Resources, Hellenic Agricultural Organization-DEMETER, in Greece.

#### Plant Material Propagation

Cultivated plants of oregano, Greek mountain tea, and thyme were propagated by cuttings (17, 18), while chamomile plants were produced by seeds (19). Cultivation was conducted during the period of 3 years (2018–2020) in the area of North-West Macedonia, Greece, from different farmers. Plants produced by cuttings were transplanted in field in the early spring of 2018. Plant density was 0.6 × 0.4 m for oregano, 0.8 × 0.6 m for Greek mountain tea and 0.8 × 0.35 m for thyme, while chamomile was sowed every year in October (400 g/acre). Harvest was conducted at full blossom (20–22) every year and post-harvest processing was undertaken by the Bagatzounis and Sons SA company, specialized on the commercialization of Greek MAPs.

The production of every farmer, every year was delivered to the company and considered as initial quantity of the lot. In particular, lot was considered for every harvest of plant material originated from the same plant species, same year of production, same farmer, same area of cultivation, and same plant material condition. The aerial parts of each plant were collected, dried, and processed in a grinding mill to separate leaves and flowers from branches. Simultaneously, the mill automatically separated the grated plant material in four grades determined according to Codex Alimentarius. The qualities A (4.5–1.0 mm), B (1.0–0.5 mm), and C (<0.5 for thyme, oregano, Greek mountain tea and <0.35 for chamomile) are commercially acceptable from the market (superior plant material) while the D quality (residual biomass) is considered non-commercial (inferior plant material).

#### Plant Sampling

The determination of the by-products percentage was conducted on samples of dry plant material of different lots during processing, in 3 years period (2018–2020). Samples were collected according to the standard sampling methods [ISO 948:1980, (23)]. The quantity of a sample that was processed (grated) depended on the quantity of initial lot. When the lot was: (i) 1–5 kg, all plant material was processed, (ii) 6–50 kg a sample of 5 kg was processed, (iii) 50–100 kg a sample of 10 kg was processed, and (iv) > 100 kg the processed sample was the square root of the quantity.

### Preparation of Extracts and EOs From Plant Material

Hydroalcoholic extracts of superior (A, B, and C grade) and inferior quality (D grade) of the collected plant material (thyme, oregano, Greek mountain tea, and chamomile) were produced using the ultrasound assistant extraction (UAE), Elma S 100 H (Elmasonic, Singen, Germany), equipped with an ultrasonic frequency of 37 kHz. In each case, 5 g of each pulverized plant material were extracted with 200 ml of a hydroalcoholic mixture (H_2_O:MeOH 50:50) in 3 consecutive circles for 30 min at 35–40°C. Solvents were evaporated under reduced pressure (*ca*. 100 mbar) using rotary evaporator (Büchi Labortechnik AG, Flawil, Switzerland) and percentage yield (w/w) for every extract was estimated. In total, 8 extracts were produced and subjected to further analysis.

The collected plant material (thyme, oregano, Greek mountain tea, and chamomile) was subjected to hydrodistillation to afford the respective EOs and aqueous extracts. For this reason, 100 g of aerial parts from the superior and inferior quality of plants were distilled using 1,000 ml water at 100°C for 3 h in a Clevenger apparatus. The percentage yield (v/w) of the produced EOs was estimated, they were dried over sodium sulfate anhydrous and stored at 4°C until they were analyzed. Furthermore, the remaining aqueous extracts were filtered, lyophilized to dryness (Zirbus Technology, Germany) and stored for further analysis.

### Chemical Evaluation of Extracts and Essential Oils

#### HPTLC Analysis

The chemical profile of the obtained extracts was determined using an HPTLC system, purchased from Camag (CAMAG, Muttenz, Switzerland). Samples were applied on silica gel F254-precoated plates from Merck (Merck, Darmstadt, Germany) using an automated sample applicator ATS4 and the chromatograms were developed in an ADC2 automated development chamber with the appropriate mobile phase. The plates were documented under UV 254 and 366 nm and after spraying with sulfuric vanillin using the TLC Visualizer 2. The system was operating under the VisionCats 2.2 and WinCats 1.4.9 software. For the HPTLC fingerprinting of hydroalcoholic and aqueous extracts, 100 μg of each sample were loaded on a normal and reversed phase TLC plate and the solvent mixture EtOAC:MeOH:A.A (70:30:1) and H_2_O:ACN:A.A (80:20:1) were used as a mobile phase, respectively.

#### Gas Chromatography-Mass Spectrometry (GC-MS) Analysis

The identification of the chemical composition of the EOs was performed with an Agilent 7820A Gas Chromatograph System linked to an Agilent 5977B mass spectrometer system (Agilent Technologies, Santa Clara, CA, USA) equipped with a HP5-MS capillary column (30 m × 0.25 mm and 0.25 μm film thickness). The initial column temperature was 60°C and then increased at a rate of 3°C/min to a maximum temperature of 300°C, where it remained for 10 min. The total analysis time was 90 min. Helium was used as a carrier gas at a flow rate of 1.0 ml/min, split ratio 1:10, injector temperature 220°C, and ionization voltage 70 eV. The compound identification was conducted using the NIST14 and ADAMS 07 libraries, bibliographic data, and the comparison of the kovats (IK) and Adams indices. The Kovats indices compare the retention time of a product with a linear alkane of the same number of carbons and were determined by injecting a mixture of alkanes (standard C9–C30) under the same operating conditions. The chromatograms were processed with Agilent MSD ChemStation Data Analysis software.

#### Ultra-High-Performance Liquid Chromatography-High-Resolution Mass Spectrometry (UHPLC-HRMS) Analysis

The ultra-high-performance liquid chromatography was performed employing a Vanquish UHPLC system (Thermo Scientific, Bremen, Germany) equipped with a binary pump, an autosampler, an online vacuum degasser, and a temperature-controlled column compartment. LC-MS grade methanol (MeOH) and formic acid (FA) were purchased from Fisher Scientific (Fisher Optima, Loughborough, UK) and LC-MS water was produced from a Barnstead MicroPure Water Purification System (Thermo Scientific, Bremen, Germany). An Accucore Vanquish UPLC C18 (2.1 mm × 50 mm, 1.5 μm) reversed phased column (Thermo Scientific, Bremen, Germany) was used for the analysis. The high-resolution mass spectrometry (HRMS) was performed on an Orbitrap Exactive Plus Mass Spectrometer (Thermo Scientific, Bremen, Germany).

Samples were prepared in duplicates and injected two times at a concentration of 100 ppm diluted in MeOH:H_2_O 50:50. The mobile phase consisted of solvents A: aqueous 0.1% (v/v) formic acid and B: acetonitrile. Different gradient elutions were performed for positive and negative ion mode detection and after optimization of the chromatography, the gradient applied was: T = 0 min, 5% B; T = 3 min, 5% B; T = 21 min, 95% B; T = 26 min, 95% B; T = 26.1 min, 5% B; and T = 30 min, 95% B. The flow rate was 0.3 ml/min and the injection volume was 5 μl. The column temperature was kept at 40°C while the sample tray temperature was set at 10°C. The ionization was performed at HESI, for both positive and negative modes. The conditions for the HRMS for both negative and positive ionization modes were set as follow: capillary temperature, 320°C; spray voltage, 2.7 kV; S-lens Rf level, 50 V; sheath gas flow, 40 arb. units; aux gas flow, 8 arb. units; aux. gas heater temperature, 50°C. The analysis was performed using the Fourier transform mass spectrometry mode (FTMS) in the full scan ion mode, applying a resolution of 70,000, while the acquisition of mass spectra was performed in every case using the centroid mode. The data dependent acquisition capability has been also used at 35,000 resolution, allowing for the tandem mass spectrometry (MS/MS) fragmentation of the three most intense ions of every peak exceeding the predefined threshold applying a 10 s dynamic exclusion. Normalized collision energy was set at 35. Data acquisition and analysis has been completed employing Xcalibur 2.1 and MZmine (24).

### DPPH Free Radical Scavenging Assay

Evaluation of the free radical scavenging activity of the produced hydroalcoholic and aqueous extracts was performed using the free radical DPPH assay as described previously (25). Extracts were prepared using DMSO as a solvent in an initial concentration of 4 mg/ml (stock solution) and dilutions were made to reach the tested concentrations (200 and 100 μg/ml). Then, 10 μl of extract in DMSO and 190 μl of DPPH solution (12.4 mg/100 ml in ethanol) were mixed in a 96-well plate and then subsequently incubated, at room temperature, for 30 min in darkness. Finally, the absorbance was measured at 517 nm in a microplate reader (Tecan, Männedorf, Switzerland). All evaluations were performed in triplicates, gallic acid was used as positive control and the percentage inhibition of the DPPH radical was estimated by the following equation:


$$\begin{array}{l} \left[\left(A-B\right)-\left(C-D\right)\right]/\left(A-B\right)\;x\;100 \end{array}$$

where A: Control (w/o sample), B: Blank (w/o sample, w/o DPPH), C: sample, D: Blank sample (w/o DPPH).

### Total Phenolic Content (TPC) Determination

The phenolic content of the extracts was determined by using a Folin–Ciocalteu colorimetric method (26). Folin–Ciocalteu solution was prepared with 10% dilution in distilled water and the alkaline environment was achieved with the addition of 7.5% sodium carbonate in distilled water. Extracts were prepared using DMSO as a solvent in stock concentrations and dilutions were made if necessary. In 96 well plates, 25 μl of extract in DMSO, 125 μl Folin–Ciocalteu solution and 100 μl Na_2_CO_3_ solution were mixed. The plates were incubated for 30 min at ambient temperature in dark. Absorbance was measured at 765 nm, using a microplate reader (Tecan, Männedorf, Switzerland). The total phenolic content (TPC) of the extracts was determined by a standard curve of absorbance values derived from standard concentration solutions of gallic acid (GA, 1.25, 2.5, 5, 10, 20, 30, 40, 50, and 100 μg/ml final concentrations). TPC was expressed as milligram of gallic acid equivalent per gram of dried extract (mg GAE/g dry weight). Each sample was tested in triplicate.

## Results

### Determination of the Percentage of MAPs By-Products

Processing of over 10 tons of oregano reveal that only 46% of the produced product was commercially acceptable, while 54% was considered as by-product (Table 1). The respective percentage for chamomile, after the processing of 0.5 tons of dried plant material was 75 and 25% by-products, for Greek mountain tea after processing of ~0.9 tons was 65 and 35% by-product and for thyme after processing of nearly 0.3 tons- was 36 and 64% by-product (Table 1). In our effort to assess MAPs by-products as a potent source of bioactive ingredients, we proceeded to the preparation of extracts and EOs from the aerial parts of superior and inferior quality of the selected herbs; thyme, oregano, Greek mountain tea, and chamomile. To this direction, eight hydroalcoholic extracts using the superior and inferior quality of the selected herbs were produced, and their percentage extraction yields were compared (samples were prepared in triplicates). Based on the results (Table 2), in the case of Greek mountain tea (MT *vs*. MTW) and chamomile (CH *vs*. CHW), both extracts of commercial and non-commercial herbal materials were characterized by similar extraction yields, whereas in the case of thyme (THV vs. THVW) and oregano (ORV vs. ORVW), by-products' extracts were characterized by lower extraction yields.

**Table 1:** Percentages of by-products derived from the cultivated Greek medicinal and aromatic plants' (MAPs) processing of different lot (between 2018 and 2000 years).

**Before processing**			**After processing**						
			**Acceptable in market**					**Non-commercially acceptable**	
**Initial plant material**	**Lot No**	**Initial weight (kg)**	**A grade (kg)**	**B grade** **(kg)**	**C grade** **(kg)**	**Total weight** **(kg)**	**Total per initial weight (%)**	**By-products (kg)**	**By- products per initial weight (%)**
*Thymus vulgaris*									
Chopped plant	8008-01	18.5	2.1	6.2	1.0	8.3	45%	10.2	55%
Chopped plant	1100-01	98.0	11.5	34.5	0.0	46.1	47%	51.9	53%
Grated plant	8049-01	164.0	0.0	0.0	24.6	24.6	15%	139.4	85%
**Total** ***Thymus vulgaris***		280.5	13.6	40.8	25.6	79.0	**36%**	201.5	**64%**
*Origanum vulgare* subsp. *hirtum*									
Whole plant	7969-1	300.0	58.8	58.8	29.4	147.0	49%	153.0	51%
Whole plant	7969-1	345.0	64.9	64.9	32.4	162.2	47%	182.9	53%
Whole plant	7969-1	300.0	58.8	58.8	29.4	147.0	49%	153.0	51%
Whole plant	7992-1	300.0	56.4	56.4	28.2	141.0	47%	159.0	53%
Whole plant	7992-1	309.0	60.6	60.6	30.3	151.4	49%	157.6	51%
Whole plant	8037-1	1,014.0	186.6	186.6	93.3	466.4	46%	547.6	54%
Whole plant	8014-01	694.0	136.0	136.0	68.0	340.1	49%	353.9	51%
Whole plant	8052-01	430.0	77.4	77.4	38.7	193.5	45%	236.5	55%
Whole plant	8037-01	1,230.0	241.1	241.1	120.5	602.7	49%	627.3	51%
Chopped plant	7976-1	650.0	96.2	96.2	48.1	240.5	37%	409.5	63%
Chopped plant	8044-01	371.0	56.4	56.4	28.2	141.0	38%	230.0	62%
Chopped plant	8092-1	1,020.0	183.6	183.6	91.8	459.0	45%	561.0	55%
Chopped plant	8121-01	17.0	3.0	3.0	1.5	7.5	44%	9.5	56%
Chopped plant	8135-01	650.0	101.4	101.4	50.7	253.5	39%	396.5	61%
Chopped plant	8139-01	1,224.0	220.3	220.3	110.2	550.8	45%	673.2	55%
Stems	8044-02	211.0	0.0	0.0	0.0	0.0	0%	211.0	100%
Stems	8135-02	350.0	0.0	0.0	0.0	0.0	0%	350.0	100%
Grated plant	8130-01	225.0	74.7	74.7	37.4	186.8	83%	38.3	17%
Grated plant	8129-01	630.0	199.1	199.1	99.5	497.7	79%	132.3	21%
Grated plant	8129-01	52.0	16.2	16.2	8.1	40.6	78%	11.4	22%
**Total** ***Origanum vulgare*** **subsp**. ***hirtum***		10,322.0	1,891.4	1,891.4	945.7	4,728.5	**46%**	5,593.5	**54%**
*Sideritis scardica*									
Grated plant	8139-04	60.0			44.4	44.4	74%	15.6	26%
Whole plant	8009-01	130.0	40.3	40.3		80.6	62%	49.4	38%
Whole plant	8009-01	175.5	42.1	42.1	21.1	105.3	60%	70.2	40%
Whole plant	002-2018	514.0	119.8	204.0		323.8	63%	190.2	37%
**Total** ***Sideritis scardica***		879.5	202.2	286.4	65.5	554.1	**65%**	325.4	**35%**
*Matricaria recutita*									
Flower/stem	7973-1	210.0	37.0	18.5	129.4	184.8	88%	25.2	12%
Flower/stem	7974-1	150.0	26.7	26.7	80.1	133.5	89%	16.5	11%
Stem	8030-01	80.0				0.0	0%	80.0	100%
Flower/stem	8113-01	40.0	9.1	5.5	21.8	36.4	91%	3.6	9%
Flower/stem	8121-02	10.0	1.3	1.5	6.4	9.2	92%	0.8	8%
Flower/stem	8139-03	50.0	6.2	7.0	30.8	44.0	88%	6.0	12%
**Total** ***Matricaria recutita***		540.0	80.2	59.2	268.5	407.9	**75%**	132.1	**25%**

**Table 2:** Percentage extraction yields of hydroalcoholic extracts deriving from superior and inferior plant material.

**Plant species**	**Plant material**	**Code**	**% extraction yield (w/w)**
*Thymus vulgaris* (thyme)	Superior	THV	28.0
	Inferior	THVW	18.6
*Origanum vulgare* subsp. *hirtum* (oregano)	Superior	ORV	30.6
	Inferior	ORVW	4.10
*Sideritis scardica* (Greek mountain tea)	Superior	MT	11.8
	Inferior	MTW	10.8
*Matricaria recutita* (chamomile)	Superior	CH	21.8
	Inferior	CHW	21.2

### Chemical Investigation and Free Radical Scavenging Activity of MAPs By-Products Extracts

#### Chemical Investigation of Hydroalcoholic Extracts

**Figure 1:**
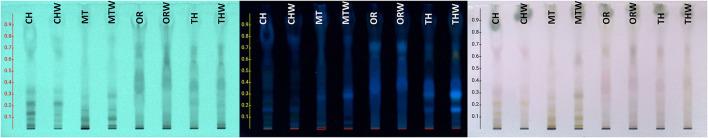
High-performance thin layer chromatography (HPTLC) of hydroalcoholic extracts of chamomile (CH, superior plant material; CHW, inferior plant material), Greek mountain tea (MT, superior plant material; MTW, inferior plant material), oregano (ORV, superior plant material; ORVW, inferior plant material), and thyme (THV, superior plant material; THVW, inferior plant material), on a reversed phase TLC plate and H_2_O:ACN:A.A: (80:20:1) as a mobile phase.

The chemical profile of hydroalcoholic extracts was investigated using HPTLC and LC-MS techniques. HPTLC chromatograms (Figure 1) revealed the presence of similar secondary metabolites in the extracts obtained from both superior and inferior raw materials for all the studied herbs. In the case of chamomile (CH *vs*. CHW) and Greek mountain tea (MT *vs*. MTW), flavonoids were detected (absorbance at 254 nm and orange/pink spots after spraying with vanillin-sulfuric acid solution and heating), phenylpropanoids (light absorbance at 254 nm and gray spots after spraying and heating), as well as sugars (no absorbance, dark gray spots after spraying and heating, increased polarity). Thyme (THV vs. THVW) and oregano extracts (ORV vs. ORVW) were mostly characterized by the presence of terpenoids (no absorbance under UV light at 254 nm and blue-purple spots after spraying and heating), flavonoids and sugars (27). The chemical content of MAP extracts as determined by LC-MS analyses is shown in Table 3. Identification of compounds was based on LC-MS data compared with the data from literature (28) and particularly, for thyme (29, 30), oregano (31, 32), Greek mountain tea (33), and chamomile (34, 35), as well as from databases, such as Dictionary of Natural Products and GNPS Public Spectral Libraries (36). Overall, the majority of identified compounds of the hydroalcoholic extracts of superior quality material were present in lower amounts or in traces in the inferior plant extracts as shown in Table 3. In particular, 29 compounds were tentatively identified for thyme, 40 for oregano, 28 for Greek mountain tea, and 31 for chamomile. The presence of phenolic acids as well as mono and disaccharides of flavonoids in the hydroalcoholic extracts of all plant species comes in agreement with literature data.

**Table 3:** Liquid chromatography-mass spectrometry (LC-MS) based characterization of *Thymus vulgaris* (thyme), *Origanum vulgare* subsp. *hirtum* (oregano), *Sideritis scardica* (Greek mountain tea), and *Matricaria recutita* (chamomile) extracts obtained from the plant material of superior and inferior quality.

**No**.	**Rt (min)**	**m/z**	**M. Formula**	**Ion mode**	**MS/MS fragments**	**Tentative identification**	**Hydroalcoholic extracts of inferior plant material**
*Thymus vulgaris* (thyme)							
1	0.41	341.1092	C12H22O11	[M-H]^−^	89, 59	bis-hexoses	y^*^
2	0.50	191.0190	C6H8O7	[M-H]^−^	111	Citric acid	y
3	0.65	359.0989	C15H20O10	[M-H]^−^	197, 179	methoxy hydroxyphenylglycol glucorinide	tr
4	3.2	305.0704	C15H14O7	[M-H]^−^	225, 97	gallocatechin	y
5	4.71	387.1667	C18H28O9	[M-H]^−^	207	hydroxyjasmonic acid hexoside	y
6	5.36	593.1522	C27H30O15	[M-H]^−^	353, 383, 473	apigenin-6,8-di-C-hexoside	y
7	6.03	447.0939	C21H20O11	[M-H]^−^	285	luteolin hexoside	y
8	6.15	355.1039	C16H20O9	[M-H]^−^	193	ferulic acid hexoside	tr
9	6.25	463.0887	C21H20O12	[M-H]^−^	300, 285	quercetin hexoside	y
10	6.3	595.1679	C27H32O15	[M-H]^−^	151, 287, 135	eriodictyol disaccharide	tr
11	6.41	461.0733	C21H18O12	[M-H]^−^	285, 300	luteolin glucuronide	y
12	6.45	447.0939	C21H20O11	[M-H]^−^	285	luteolin hexoside	y
13	6.65	521.1306	C24H26O13	[M-H]^−^	323, 161, 179	rosmarinic acid hexoside	y
14	6.82	193.0500	C10H10O4	[M-H]^−^	161, 137	ferulic acid	y
15	6.88	471.1876	C22H32O11	[M-H]^−^	165, 99,	Unidentified	y
16	6.90	553.0997	C27H22O13	[M-H]^−^	135, 161, 179	Caffeoyl feruloylquinic acid	y
17	6.93	445.0778	C21H18O11	[M-H]^−^	269	apigenin glucuronide	y
18	7.14	359.0779	C18H16O8	[M-H]^−^	161, 197	rosmarinic acid	y
19	7.2	555.1151	C27H24O13	[M-H]^−^	161, 135, 197	salvianolic acid derivative (K)	y
20	7.26	493.1149	C26H22O10	[M-H]^−^	161, 135, 197	salvianolic acid derivative (A)	y
21	7.34	371.1352	C17H24O9	[M-H]^−^	191	sinapyl alcohol monosaccharide derivative (syringin)	tr
22	7.36	717.1482	C36H30O16	[M-H]^−^	357, 283, 339	salvianolic acid derivative (B)	y
23	7.44	315.0515	C16H12O7	[M-H]^−^	300	tetrahydroxy-7-methoxyflavone	y
24	7.50	537.1047	C27H22O12	[M-H]^−^	197, 239, 137	salvianolic acid derivative (H, I)	y
25	7.93	373.0934	C19H18O8	[M-H]^−^	135, 175, 197, 160	rosmarinic acid methylester	y
26	7.98	285.0409	C15H10O6	[M-H]^−^		kaempferol/luteolin	y
27	8.18	299.0564	C16H12O6	[M-H]^−^	284	methyl kaempferol/ methyl luteolin	y
28	8.65	271.0617	C15H12O5	[M-H]^−^	151, 119	naringenin	y
29	9.25	327.2180	C18H32O5	[M-H]^−^	211, 229, 183	trimethoxy hydroxyflavone	y
*Origanum vulgare* subsp. *hirtum* (oregano)							
30	0.41	179.0553	C6H12O6	[M-H]^−^	75	hexoses	y
31	0.46	341.1092	C12H22O11	[M-H]^−^	89, 59	bis-hexose	y
32	0.42	191.0554	C7H12O6	[M-H]^−^	111	quinic acid	y
33	0.52	191.019	C6H8O7	[M-H]^−^	111	Citric acid	y
34	0.77	197.0449	C9H10O5	[M-H]^−^	72, 135	syringic acid	y
35	4.82	387.1633	C18H28O9	[M-H]^−^	207, 163	hydroxyjasmonic acid hexoside	y
36	4.91	609.0897	C27H30O16	[M-H]^−^	369, 399, 489	luteolin disaccharide	y
37	5.3	593.1521	C27H30O15	[M-H]^−^	353, 383, 474	apigenin disaccharide	y
38	5.55	637.1058	C27H26O18	[M-H]^−^	285, 113, 351	luteolin diglucuronide	y
39	5.78	447.094	C21H20O11	[M-H]^−^	327, 357, 285	luteoline hexoside	y
40	6.01	621.1106	C27H26O17	[M-H]^−^	113, 269, 285. 193	apigenin diglucuronide	y
41	6.22	431.0988	C21H20O10	[M-H]^−^	311, 283	apigenin hexoside	y
42	6.3	799.1382	C36H32O21	[M-H]^−^	285, 179, 135, 351	unidentified	y
43	6.38	461.0733	C21H18O12	[M-H]^−^	285, 300	luteolin glucuronide	y
44	6.49	715.1321	C36H28O16	[M-H]^−^	321, 295, 339	unidentified	y
45	6.66	537.1046	C27H22O12	[M-H]^−^	295, 399	salvianolic acid derivative (J/I/H)	y
46	6.69	717.1475	C36H30O16	[M-H]^−^	339, 243, 135	salvianolic acid derivative (E/B)	y
47	6.89	719.1634	C36H32O16	[M-H]^−^	169, 197	salvianolic acid derivative	y
48	6.93	445.0782	C21H18O11	[M-H]^−^		apigenin glucuronide	y
49	7.01	717.147	C36H30O16	[M-H]^−^	313, 295, 321	salvianolic acid derivative (E/B)	y
50	7.1	359.0773	C18H16O8	[M-H]^−^	161, 197	rosmarinic acid isomer	y
51	7.2	493.1149	C26H22O10	[M-H]^−^	109, 185, 295	salvianolic acid derivative (A)	y
52	7.28	537.1046	C27H22O12	[M-H]^−^	321, 295, 339	salvianolic acid derivative (J/I/H)	y
53	7.57	717.1475	C36H30O16	[M-H]^−^	321, 295	salvianolic acid derivative (E/B)	y
54	7.73	343.0827	C18H16O7	[M-H]^−^	145, 197	dihydroxy trimethoxy flavone	y
55	7.79	287.0565	C15H12O6	[M-H]^−^	151	eriodictyol	y
56	7.89	491.0991	C26H20O10	[M-H]^−^	311	salvianolic acid derivative (C)	y
57	7.95	285.0409	C15H10O6	[M-H]^−^	151	luteolin	y
58	8.05	717.1477	C36H30O16	[M-H]^−^	339, 321	salvianolic acid derivative (iso E/B)	y
59	8.13	299.0564	C16H12O6	[M-H]^−^	284	trihydroxy methoxy flavone (methyl kaempferol)	y
60	8.19	493.1148	C26H22O10	[M-H]^−^	109, 295, 185	salvianolic acid derivative (A)	y
61	8.39	329.0671	C17H14O7	[M-H]^−^	314, 299	trihydroxy dimethoxy flavone	y
62	8.49	373.0933	C19H18O8	[M-H]^−^	135, 175, 197	rosmarinic acid methyl ester	y
63	8.64	271.0615	C15H12O5	[M-H]^−^	151	trihydroxyflavanone (naringenin)	y
64	8.67	329.0671	C17H14O7	[M-H]^−^	299, 314	trihydroxy dimethoxy flavone	y
65	8.72	269.046	C15H10O5	[M-H]^−^	151, 119	apigenin	y
66	8.74	717.1478	C36H30O16	[M-H]^−^	339, 311, 353	salvianolic acid derivative	y
67	8.93	359.0778	C18H16O8	[M-H]^−^	329, 344	jaseidin isomer	y
68	9.25	327.218	C18H32O5	[M-H]^−^	211, 229	trihydroxy octadecadienoic acid	y
69	9.96	313.0722	C17H14O6	[M-H]^−^	283, 298	dimethoxy dihydroxyflavone	y
*Sideritis scardica* (Greek mountain tea)							
70	0.3	162.0528	C6H10O5	[M-H]^−^		polysacharides residues	y
71	0.41	341.1089	C12H22O11	[M-H]^−^	89, 59	bis-hexose	y
72	0.45	191.0552	C7H12O6	[M-H]^−^	111	quinic acid	y
73	1.8	353.0882	C16H18O9	[M-H]^−^	191	Caffeoylquinic acid derivative	y
74	5.33	435.1512	C16H24O10	[M+Hac-H]	341, 321	fatty acyl dissaccharide	y
75	6.19	625.1414	C27H30O17	[M-H]^−^	301, 445	hypolaetin disaccharide	y
76	6.29	785.2523	C35H46O20	[M-H]^−^	179, 161	phenylethanoid trisaccharide (echinacoside)	y
77	6.31	521.2034	C26H34O11	[M-H]^−^	329	dihydrodehydrodiconiferyl alcohol hexoside	y
78	6.4	667.1528	C29H32O18	[M-H]^−^	301, 139	hypolaetin acetyl-hexoside	y
79	6.46	755.2416	C34H44O19	[M-H]^−^	161, 461	phenylethanoid trisaccharide (lavandulifolioside)	y
80	6.55	623.1955	C29H36O15	[M-H]^−^	161, 113, 461	phenylethanoid disaccharide (verbascoside isomer)	y
81	6.63	609.1471	C27H30O16	[M-H]^−^	447	isoscutellarein disaccharide	y
82	6.75	623.1991	C29H36O15	[M-H]^−^	161, 299, 284	phenylethanoid disaccharide (verbascoside isomer)	y
83	6.87	431.0983	C21H20O10	[M-H]^−^	268	apigenin hexoside	y
84	6.91	641.1722	C28H32O17	[?+?]^+^	317	tetrahydroxy flavone dissacharide	y
85	6.98	769.2564	C35H46O19	[M-H]^−^	161, 175	phenylethanoid disaccharide (sideritiside)	y
86	7.12	637.2148	C30H38O15	[M-H]^−^	175, 160	phenylethanoid disaccharide (leucoseptoside)	y
87	7.32	651.1579	C29H32O17	[M-H]^−^	285	isoscutellarein acetyl dissacharide	y
88	7.54	681.1683	C30H34O18	[M-H]^−^	315, 300	methyl hupolaetin acetyl dissacharide	y
89	7.83	623.1627	C28H32O16	[M-H]^−^	299, 284, 161	methyl isoscutellarein dissacharide	y
90	8.57	577.1359	C30H26O12	[M-H]^−^	269	apigenin coumaroyl hexoside	y
91	8.6	665.1735	C30H34O17	[M-H]^−^	299	methyl isoscutellarein acetyl dissacharide	y
92	8.65	269.0459	C15H10O5	[M-H]^−^	151	apigenin	y
93	8.77	723.1796	C32H36O19	[M-H]^−^	315, 300	methyl hupolaetin diacetyl dissacharide	y
94	9.1	577.1359	C30H26O12	[M-H]^−^	269	apigenin coumaroyl hexoside	y
95	9.79	707.1841	C32H36O18	[M-H]^−^	299, 284	methyl isoscutellarein diacetyl dissacharide	y
96	10.55	343.0822	C18H16O7	[M-H]^−^	313, 328	dihydroxy trimethoxyflavone	y
97	11.76	395.2442	C22H35O6	[M-H]^−^	165, 90	sideripullol derivative	
*Matricaria recutita* (chamomile)							
98	0.41	179.0553	C6H12O6	[M-H]^−^	75	hexoses	y
99	0.48	191.019	C6H8O7	[M-H]^−^	111	Citric acid	y
100	0.50	341.1092	C12H22O11	[M-H]^−^	89, 59	bis-hexoses	y
101	2.01	353.0881	C16H18O9	[M-H]^−^	191	Chlorogenic acid	y
102	4.38	355.1039	C16H20O9	[M-H]^−^	149, 193	hydroxy methoxycinnamic acid hexoside isomer 1	y
103	5.45	323.0776	C15H16O8	[M-H]^−^	121	umbellliferone hexoside	y
104	5.48	367.1036	C17H20O9	[M-H]^−^	93, 173	feruloylquinic acid	y
105	5.55	479.0837	C21H20O10	[M-H]^−^	317, 165	myricetin hexoside	y
106	5.75	337.0933	C16H18O8	[M-H]^−^	87, 219, 201	daphnetin hexoside	y
107	5.80	463.0889	C21H20O12	[M-H]^−^	301, 151	quercetin hexoside 1	y
108	5.92	355.1039	C16H20O9	[M-H]^−^	149, 193	hydroxy methoxycinnamic acid hexoside isomer 2	y
109	6.20	463.0885	C21H20O12	[M-H]^−^	301, 151	quercetin hexoside 2	y
110	6.30	593.1521	C27H30O15	[M-H]^−^	285	luteolin disaccharide	y
111	6.37	447.0938	C21H20O11	[M-H]^−^	285	luteolin hexoside	y
112	6.49	493.0994	C22H22O13	[M-H]^−^	331, 168, 316	patuletin hexoside	y
113	6.65	515.1199	C25H24O12	[M-H]^−^	191, 179	dicaffeylquinic acid derivative	y
114	6.75	515.1198	C25H24O12	[M-H]^−^	191, 179	dicaffeylquinic acid derivative	y
115	6.79	577.1563	C27H30O15	[M-H]^−^	269	apigenin disaccharide	y
116	6.92	431.0989	C21H20O10	[M-H]^−^	268	apigenin hexoside	y
117	7.09	479.1189	C22H22O12	[?+?]^+^	317	isorhamnetin hexoside	y
118	7.09	515.1198	C25H24O12	[M-H]^−^	173, 179, 191	dicaffeylquinic acid derivative	y
119	7.52	517.1357	C24H22O13	[M-H]^−^	271	apigenin malonylhexoside	tr
120	7.61	473.1094	C23H22O11	[M-H]^−^	286	apigenin acetylhexoside	tr
121	7.74	473.1096	C23H22O11	[M-H]^−^	286	apigenin acetylhexoside	tr
122	8.00	593.1313	C30H26O13	[M-H]^−^	269	apigenin caffeylhexoside	tr
123	8.06	517.1356	C25H26O12	[M-H]^−^	269	apigenin malonylhexoside	tr
124	8.27	473.1088	C23H22O11	[M-H]^−^	473, 268	apigenin acetyl- malonyl- hexoside	tr
125	8.39	515.1200	C25H24O12	[M-H]^−^	173, 179, 191	dicaffeylquinic acid derivative	y
126	8.68	269.0458	C15H10O5	[M-H]^−^	151	trihydroxyflavone	tr
127	9.54	305.1385	C17H20O5	[M+H]^+^	245	sesquiterpene lactone (matricarin)	tr
128	10.26	373.0928	C19H18O8	[M-H]^−^		dihydroxy tetramethoxyflavone	y

* *y = presence of compound in the inferior plant material extracts, tr = traces of the identified compound in the inferior plant material extracts*.

*Annotation table was constructed based on compounds present in superior plant material extracts*.

#### Evaluation of TPC and Free Radical Scavenging Activity of Hydroalcoholic Extracts

The TPC of plant extracts, measured by Folin–Ciocalteu method ranged from 20.3 to 177.2 mg GAE/g dry weight (Table 4) with the highest phenolic content found in thyme and oregano extracts. More specifically in the case of thyme, both the categories of plant material were characterized by similar levels of phenols (THV:177.2 mg GAE/g dw, and THW:166.4 mg GAE/g dw), followed by oregano extracts where no statistically significant differences were detected (ORV:160.1 mg GAE/g dw, and ORVW:143.8 mg GAE/g dw). The phenolic content of Greek mountain tea extracts ranged from 58 (MT) to 68.3 (MTW) mg GAE/g dw, while chamomile was characterized by the lowest phenolic content (<35 mg GAE/g dw) for both superior (CH) and inferior (CHW) plant material.

**Table 4:** Total phenolic content (TPC) and antioxidant capacity of hydroalcoholic extracts.

**Plant species**	**Plant material**	**Code**	**% DPPH inhibition**		**TPC**
			**200 μg/mL**	**100 μg/mL**	**mg GAE/g dry weight**
*Thymus vulgaris* (thyme)	Superior	THV	85.9 ± 0.6	66.6 ± 0.8	177.2 ± 8.2
	Inferior	THVW	82.7 ± 0.7	78.6 ± 2.7	166.4 ± 4.7
*Origanum vulgare* subsp. *hirtum* (oregano)	Superior	ORV	91.5 ± 0.1	87.1 ± 0.2	160.1 ± 8.0
	Inferior	ORVW	78.1 ± 1.0	78.1 ± 1.0	143.8 ± 7.0
*Sideritis scardica* (Greek mountain tea)	Superior	MT	74.8 ± 5.9	40.9 ± 3.3	68.3 ± 8.4
	Inferior	MTW	58.2 ± 2.4	29.0 ± 0.3	58.0 ± 2.0
*Matricaria recutita* (chamomile)	Superior	CH	66.6 ± 1.0	38.2 ± 5.0	34.9 ± 2.9
	Inferior	CHW	28.3 ± 1.6	13.1 ± 0.8	20.3 ± 3.1

The DPPH inhibition at 200 μg/ml final concentration was stronger for oregano (78.1% ORVW−91.5% ORV) and thyme (82.7% THVW−85.9% THV), with minor differences detected among the different plant material (Table 4). Greek mountain tea extract was characterized as a potent antioxidant factor (37), in comparison with literature (10), at 200 μg/ml (MT:74.8% inhibition) whereas its by-product extract, revealed slightly reduced activity at the same concentration (MTW: 58.2% inhibition). Finally, chamomile extracts, exhibited moderate antioxidant activity. It is noteworthy, that the comparison between DPPH and TPC methods, revealed strong correlations (*r* 0.8177, *p* < 0.013) between phenolic content and scavenging properties in all tested extracts.

Based on the above results, the primary evaluation of oregano, thyme, Greek mountain tea, and chamomile by-products revealed similar chemical content to the superior herbal material and strong free radical scavenging capacity related to a high phenolic content. Therefore, since they constitute a very promising source of bioactive compounds, further investigation regarding their potent exploitation was followed.

### Chemical Investigation and Evaluation of EOs and Aqueous Extracts

Taking into consideration the main use of thyme and oregano for culinary purposes, their EOs were produced *via* hydrodistillation to evaluate the volatile content of the herbs. As expected, oregano afforded the best yield in EO production (ORV_HDEO: 4%, ORVW_HDEO: 0.8 %) followed by thyme (THV_HDEO: 1.2%, THVW_HDEO: 0.15%). Results are shown in Table 5. It is worth noticing that in both cases the by-products afforded even a small percentage of EO. In the case of chamomile only superior quality (CH_HDEO) afforded 0.4% EO, whereas Greek mountain tea did not produce EO at all. However, considering the wide use of Greek mountain tea and chamomile as infusions, the remaining aqueous extracts of superior (MT_HDAQ and CH_HDAQ) and inferior (MTW_HDAQ and CHW_HDAQ) qualities from the hydrodistillation process were selected for further evaluation, regarding their chemical content and free radical scavenging activity.

**Table 5:** Percentage yields of essential oil and aqueous extracts deriving from superior and inferior plant material.

**Plant species**	**Plant material**	**Code**	**% EO yield**	**Code**	**% extraction yield (v/w)**
*Thymus vulgaris* (thyme)	Superior	THV_HDEO	1.2		15.3
	Inferior	THVW_HDEO	0.15		6.0
*Origanum vulgare* subsp. *hirtum* (oregano)	Superior	ORV_HDEO	4.0		6.1
	Inferior	ORVW_HDEO	0.8		5.6
*Sideritis scardica* (Greek mountain tea)	Superior		-	MT_HDAQ	13.7
	Inferior		-	MTW_HDAQ	13.5
*Matricaria recutita* (chamomile)	Superior	CH_HDEO	0.4	CH_HDAQ	12.1
	Inferior		-	CHVW_HDAQ	12.6

#### GC-MS Analysis of EO of Thyme and Oregano

Based on the results of GC-MS analyses, in total 20 constituents were identified in oregano superior plant material (ORV_HDEO) representing 99.98% of the total content (Table 6), 14 of which were detected in the inferior quality as well (ORVW_HDEO). The major constituents of oregano EO were carvacrol, thymol, *p*-cymene, and γ-terpinene which were detected in both plant materials in corresponding amounts. Especially in the case of carvacrol—which was found to be the predominant constituent—and *p*-cymene, their percentage in the inferior plant material were slightly higher (78.20 and 6.68%, respectively) compared with the superior plant material (64.78 and 4.29%, respectively). Other compounds present in oregano by-product were: δ-2-carene, β-myrcene, terpinen-4-ol, *trans-*caryophyllene, borneol, caryophyllene oxide, β-phellandrene, and *trans*-sabinene hydrate. Surprisingly, eugenol was detected only in the by-product in a percentage of 0.21%.

**Table 6:** Chemical composition of the essential oils (EOs) of superior and inferior plant material of *T. vulgaris* (thyme) and *O. vulgare* subsp. *hirtum* (oregano).

		***Origanum vulgare*** **subsp**. ***hirtum***		***Thymus vulgaris***	
		**Superior (ORV_HDEO)**	**Inferior (ORVW_HDEO)**	**Superior (THV_HDEO)**	**Inferior (THVW_HDEO)**
**KI**	**Constituents**	**Area %**			
952	α-pinene	0.40	-	0.90	-
962	camphene	0.09	-	0.77	-
981	1-octen-3-ol	0.25	-	0.60	-
992	β-myrcene	0.57	0.65	0.34	0.44
1003	α-phellandrene	0.09	0.10	-	-
1014	δ-2-carene	0.71	0.72	0.79	0.68
1022	*p*-cymene	4.29	6.68	22.50	21.88
1025	limonene	-	-	0.33	0.21
1025	β-phellandrene	0.23	0.28	-	-
1027	1,8-cineol	-	-	0.61	0.43
1055	γ-terpinene	4.00	2.13	4.43	2.00
1070	*trans*-sabinene hydrate	0.22	0.17	0.15	0.16
1088	terpinolene	-	-	0.15	0.12
1099	linalool	0.13	-	1.30	1.62
1138	camphor	-	-	0.51	1.73
1160	borneol	0.39	0.30	1.71	1.69
1173	terpinen-4-ol	0.47	0.61	0.66	0.72
1186	α-terpineol	-	-	0.22	0.23
	thymol methyl ether	-	-	1.23	1.36
1239	carvacrol methyl ether	0.27	-	0.84	0.40
1291	thymol	20.14	7.19	53.04	56.50
1306	carvacrol	64.78	78.20	5.15	7.25
1351	eugenol	-	0.21	0.12	0.09
1412	*trans*-caryophyllene	1,26	0.80	1.07	0.86
1446	α-humulene	0.16	0.10	-	-
1471	Trans-muurola-3,5-diene	-	-	0.09	-
1472	Geranyl propanoate	-	-	0.12	-
1505	β-bisabolene	1.19	-	-	-
1507	γ-cadinene	-	-	0.18	-
1573	caryophyllene oxide	0.34	0.43	1.33	-
Total %	99.98	98.57	99.14	98.37

*Oregano (ORV_HDEO, superior plant material; ORVW_HDEO, inferior plant material) and thyme (THV_HDEO, superior plant material; THVW_HDEO, inferior plant material)*.

Correspondingly, in the case of the chromatographic analyses of thyme EO, 26 constituents were identified in thyme superior plant material (THV_HDEO) representing 99.14% of the total content (Table 6), 19 of which were detected in the inferior quality as well (THVW_HDEO). The major constituents detected were thymol (>50%), *p*-cymene (>20%), carvacrol (5–7%), γ-terpinene (2.0–4.4%), linalool (1.3–1.6%), and borneol (1.7%), and were present in both plant materials in similar amounts. In this case, thymol was found to be the predominant constituent of thyme followed by *p*-cymene, with slightly higher percentages detected in the inferior plant material compared with the superior, as depicted in Table 6. Other common constituents were carvacrol methyl ether, thymol methyl ether, trans-caryophyllene, β-myrcene, limonene, δ-2-carene, 1,8-cineol, and camphor, terpinen-4-ol. On the other hand, α-pinene, camphene and 1-octen-3-ol were only detected in the inferior quality of both studied herbs.

The remaining aqueous extracts from the hydrodistillation process of Greek mountain tea (MTW_HDAQ) and chamomile (CHW_HDAQ) by-products, were chemically investigated using HPTLC and LC-MS techniques. The aqueous extracts of superior and inferior plant material were lyophilized and no significant differences were noted regarding their percentage extraction yield (Table 5). Their chemical profile was investigated using HPTLC and LC-MS techniques. The results revealed that all by-products extracts showed identical chemical profile compared with the superior quality extracts, characterized by the presence of phenolic compounds, flavonoids, and sugars. Analysis by LC-MS confirmed the similar profile of aqueous extracts of superior and by-products material. However, their chemical content was not as rich as the hydroalcoholic ones. In particular, Greek mountain tea (MT_HDAQ, MTW_HDAQ) was rich in phenylethanoid disaccharides and more specifically compounds 74, 79, 80, 82, 83, and 86–91 (Table 3) were detected, while in the case of chamomile (CH_HDAQ, CHW_HDAQ) cinnamic acid, caffeoylquinic acid derivatives were present along with some flavone and flavonol derivatives; compounds 102, 105, 108, 116–118, 124, and 125 were detected as shown in Table 3.

#### Evaluation of TPC and Free Radical Scavenging Activity

All extracts were characterized by the similar levels of phenols ranging from 12.4 to 21.5 mg GAE/g dw while not statistically significant differences (*p* > 0.3260 for Greek mountain tea and *p* > 0.2655 for chamomile) were detected between superior and inferior plant material in both cases (Table 7). Regarding the free radical scavenging activity, Greek mountain tea extract was characterized as a moderate antioxidant factor at 200 g/ml concentration (MT_HDAQ: 31.4% inhibition) whereas its by-product extract, revealed slightly increased activity at the same concentration (MTW_HDAQ: 55.3% inhibition). Finally, chamomile extracts (CH_HDAQ and CHW_HDAQ) exhibited low antioxidant activity. The reduced free radical scavenging activity and the lower phenolic content exhibited by the aqueous extracts compared with the hydroalcoholic ones, are attributed to the lower chemical profile as described above.

**Table 7:** The TPC and antioxidant capacity of hydroalcoholic extracts.

**Plant species**	**Plant material**	**Code**	**% DPPH Inhibition 200 μg/mL**	**TPC** **mg GAE/g dry weight**
*Sideritis scardica* (Greek mountain tea)	Superior	MT_HDAQ	31.4 ± 2.5	14.8 ± 0.5
	Inferior	MTW_HDAQ	55.3 ± 0.6	21.5 ± 1.8
*Matricaria recuita* (chamomile)	Superior	CH_HDAQ	12.94 ± 1.4	14.4 ± 0.8
	Inferior	CHW_HDAQ	7.5 ± 1.2	12.3 ± 0.3

## Discussion

The goal of this research was to compare the chemical content and antioxidant activity, as well as to value the potent exploitation of the current post-harvest processing by-products toward the development of innovative “food products.” The presence of phenolic acids, as well as mono- and disaccharides of flavonoids in the hydroalcoholic and aqueous extracts of all plant species comes in agreement with literature data. Moreover, evidence of the presence of these substances in the respective by-products extracts, justifies the high TPC and free scavenging activity as determined by the DPPH assay. Hence, inferior plant material not intended for the market could be utilized for the production of instant beverages. In addition, the hydroalcoholic extracts of inferior plant material and aqueous extracts remaining after hydrodistillation can serve as a source of bioactive ingredients to fortify food products and supplements. The EOs of aromatic plants, especially from thyme and oregano, are used as food additives due to their antibacterial properties. In this study, it is evident that the presence of thymol and carvacrol in the EOs of by-products, known for their antimicrobial activity (38). Hence, thyme and oregano by-products could be exploited as food antimicrobial additives, due to their potential bacteriostatic activity. Moreover, the infusion of oils with aromatic plants has proven to increase their oxidative stability and shelf-life (39). Hence, the presence of terpenes and other volatile constituents in the studied inferior plant material could be further exploited for the production of enriched aromatic edible oils and especially of functional olive oils.

In conclusion, taking into consideration all the aforementioned results, it is obvious that the non-commercially acceptable plant material is a valuable source of bioactive compounds and could be further exploited as food antimicrobial and/or antioxidant additives, for the production of innovative nutritional products, such as herbal instant beverages or enriched aromatic olive oils.

## Data Availability Statement

The original contributions presented in the study are included in the article/supplementary material, further inquiries can be directed to the corresponding author.

## Author Contributions

IG, KG, and NA conceived and designed the experiment. ED, AV, AC, PB, EA, and KG collected raw materials. ED, AV, AC, KG, and NA performed the analytical experiments. ED, AV, AC, and NA analyzed the data and interpreted the results. ED, AV, AC, IG, KG, and NA wrote the manuscript. All authors contributed to the article and approved the submitted version.

## Funding

This research has been co-financed by Greece and the European Union (European Regional Development Fund) in context Research—Create—Innovate within the Operational Program Competitiveness, Entrepreneurship, and Innovation (EPANEK) of the NSRF 2014-2020. Project Code: T1EΔK-05041. Acronym FeedMAP.

## Conflict of Interest

PB was employed by Bagatzounis & Sons S.A. EA was employed by ELVIZ Hellenic Feedstuff Industry S.A. The remaining authors declare that the research was conducted in the absence of any commercial or financial relationships that could be construed as a potential conflict of interest.

## Publisher's Note

All claims expressed in this article are solely those of the authors and do not necessarily represent those of their affiliated organizations, or those of the publisher, the editors and the reviewers. Any product that may be evaluated in this article, or claim that may be made by its manufacturer, is not guaranteed or endorsed by the publisher.
